# Enhanced Mechanical Performance of Fluoroelastomer Composites with Boron–Gadolinium-Based Fillers for Cutting-Edge Applications

**DOI:** 10.3390/polym18010006

**Published:** 2025-12-19

**Authors:** Allan Bascuñan-Heredia, Francisco Molina, Maria José Inestrosa-Izurieta, Mohamed Dahrouch, Adolfo Henriquez, Catalina Cortés, Miguel Angel Lopez-Manchado, Héctor Aguilar-Bolados

**Affiliations:** 1 Departamento de Polímeros, Facultad de Ciencias Químicas, Universidad de Concepción, Concepción 3349001, Chile; abascunan2017@udec.cl; 2 Centro de Investigación en Física Nuclear y Espectroscopia de Neutrones CEFNen, Comisión Chilena de Energía Nuclear, Santiago 7600713, Chile; francisco.molina@cchen.cl; 3 Millennium Institute for Subatomic physics at High Energy Frontier (SAPHIR), Santiago 7591538, Chile; 4 Centro de Materiales para la Transición y Sostenibilidad Energética, Comisión Chilena de Energía Nuclear, Santiago 7600713, Chile; mariajose.inestrosa@cchen.cl; 5 Departamento de Química Orgánica, Facultad de Ciencias Químicas, Universidad de Concepción, Concepción 3349001, Chile; mdahrouch@udec.cl; 6 Departamento de Química Analítica e Inorgánica, Facultad de Ciencias Químicas, Universidad de Concepción, Concepción 3349001, Chile; adohenriquez@udec.cl; 7 Departamento de Química, Facultad de Ciencias, Universidad de Chile, Santiago 7800003, Chile; catalinacortes89@gmail.com; 8 Instituto de Ciencia y Tecnología de Polímeros (ICTP), CSIC, Juan de la Cierva, 3, 28006 Madrid, Spain

**Keywords:** fluoropolymers, elastomers, inorganic materials, mechanical properties

## Abstract

The notable interest in materials with high-performance multifunctional properties, coupled with the diverse availability of raw materials—despite geopolitical controversies—allows for the design of a wide variety of new materials. Flexible materials with inorganic fillers derived from rare earths are of particular interest, as elements such as gadolinium have multiple properties of high technological interest. In particular, gadolinium oxides and borates have not been explored as fillers in special rubbers, such as FKM fluoroelastomers, which correspond to copolymers based on hexafluoropropylene and difluorovinylidene. It is in this context that the present work consists of obtaining and characterizing FKM-based compounds containing gadolinium(III) oxide, gadolinium borate, or thermally treated gadolinium borate. The promising results allow us to identify unique qualities imparted by this type of filler in fluoroelastomers, especially regarding mechanical properties. In fact, the increase in tensile strength of the compounds reached up to 162%. Likewise, the elongation at break was almost doubled. DMA identified that the reinforcing effect of gadolinium compounds is limited; it is hypothesized that the electronic nature of gadolinium, with its available f orbitals, influences the structure of FKM and, consequently, its properties. Taken together, these results provide relevant information for the development of new materials that, due to their boron and gadolinium-based composition—both elements with high neutron capture cross sections—could be used in neutron shielding applications.

## 1. Introduction

Elastomers are versatile polymeric materials that are indispensable in modern life, with applications ranging from technology and industry to the domestic sphere. This ubiquity is due to their unique mechanical properties, such as flexibility, viscoelasticity, and ease of processing [1,2]. Among them, fluoroelastomers, classified as FKM by the ASTM [3], stand out. These are random copolymers based on hexafluoropropylene and difluorovinylidene. These materials are not only notable for their superior mechanical properties and wide service temperature range, but also for their greater ability to attenuate gamma radiation compared to conventional elastomers [4,5].

To improve their performance in specific applications, such as radiation protection, the incorporation of inorganic fillers into elastomeric matrices has been extensively researched. Current research in this field has focused mainly on the use of fillers with a high atomic number (Z), such as bismuth oxide (Bi_2_O_3_) or lead oxide (PbO), achieving significant increases in density and, therefore, in gamma ray attenuation [6,7,8,9]. However, these approaches have inherent limitations, such as deterioration of mechanical properties at high filler levels, problems with dispersion and homogenization of the filler within the polymer matrix, and, in some cases, environmental and toxicological considerations. At the same time, there is growing interest in exploring rare earth materials as functional fillers due to their magnetic and optical properties, as well as their ability to modulate mechanical properties. For example, lanthanum trimethacrylate (La-TMA) has been reported to significantly increase tensile strength and elongation in PP/EPDM matrices, demonstrating the potential of these elements to reinforce elastomers beyond their radiation-shielding function [10]. The preparation of silicone elastomers with rare earths (Eu_2_O_3_, Gd_2_O_3_, and Dy_2_O_3_) has also been reported, which exhibited luminescence properties and changes in their dielectric properties [11].

Among the rare earths, gadolinium is of particular interest for applications that interact with ionizing radiation, as it has the highest thermal neutron capture cross section of all stable elements (σ_a = 49,700 barn) [12,13]. Although gadolinium compounds have demonstrated good performance in various applications, such as lighting, electronics, and magnetism [14,15,16,17,18,19,20], their systematic incorporation as fillers in advanced elastomers, and more specifically in fluoroelastomers, is an under-explored area of research. Furthermore, the synergistic combination of gadolinium with another element with high neutron capture, such as boron, to design hybrid fillers (e.g., gadolinium borates) for flexible elastomeric matrices represents an innovation with multifunctional potential that has not been exhaustively documented in recent scientific literature.

In this context, the present research addresses the following knowledge gaps: (i) the lack of studies on the effect of oxides, and, in particular, gadolinium borates, on the thermo-mechanical and dynamic-mechanical properties of fluoroelastomers; and (ii) the need to develop synthesis and dispersion methodologies for these new hybrid fillers. The innovation of this work lies in the design, synthesis by coprecipitation from gadolinium (III) oxide and sodium borate, and comprehensive characterization of three types of fillers (gadolinium oxide, gadolinium borate, and heat-treated gadolinium borate) within an FKM. The main objective is to establish manufacturing criteria and systematically evaluate whether the incorporation of these gadolinium- and boron-based fillers compromises or improves the inherent properties of the matrix, analyzing not only their possible effect on radiation interaction, but also their impact on the mechanical, dynamic-mechanical, thermal, and structural properties of the final composite.

## 2. Materials and Methods

### 2.1. Materials

The fluoroelastomer, vinylidene fluorine and hexafluoropropylene (DFV/HFP) used was FKM C60 supplied by Jiangsu FreChem Co., Ltd. (Nanjing, China). Gadolinium oxide nanoparticles, GdO (99.9%, 90–120 nm particle size), gadolinium nitrate hexahydrate, boric acid, sodium hydroxide, magnesium oxide (MgO), calcium hydroxide (Ca(OH)_2_), 2,2-bis(4-hydroxyphenyl) hexafluoropropane (Bisphenol AF), and benzyl triphenylphosphonium chloride (BPP) and ethanol (99.9%) were purchased from Sigma Aldrich (St. Louis, MO, USA).

### 2.2. Gadolinium-Based-Materials Preparation

Gadolinium borate was prepared by mixing an acidic solution (pH = 1) of 0.1 M gadolinium nitrate hexahydrate and a solution of 0.1 M boric acid. Both solutions were mixed in equal parts under constant stirring at room temperature. The solution was then brought to pH = 7 by slowly adding 0.1 M sodium hydroxide. When a pH close to 7 was reached, a white precipitate formed, which was separated from the supernatant and left to dry for 12 h at 105 °C. Once dry, the sample was divided into two equal parts. One part was set aside and labeled GdB. The other part was subjected to heat treatment consisting of a heating ramp of 5 °C/min to 400 °C, maintaining this temperature for 4 h. After this time, the sample was allowed to cool and was labeled GdBT.

### 2.3. FKM-Based Composites Preparation

**Figure 1:**
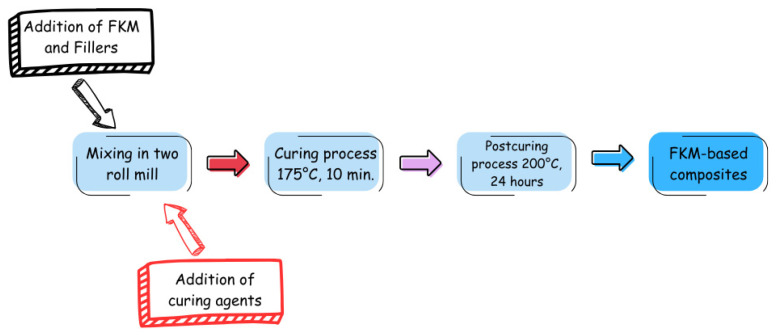
Schematic diagram of the composite preparation.

The powder materials GdO, GdB and GdBT were used as fillers in FKM fluoroelastomer. The mixing was carried out using a ZL-3018 two-roll mill from Zhongli Instrument Technology Co. Ltd. (Dongguan, China), the Figure 1 present a schematic diagram of the composites preparation. To carry out the mixing, 100 g of FKM C60 were processed for 5 min to achieve a homogeneous tape in the two rolls, with a gap of 1 mm between them. Then, determined amounts of magnesium(II) oxide and calcium(II) hydroxide, followed by the filler, were added slowly, and the mixture was processed for 20 min to achieve homogeneous dispersion. After that, the curing agents, bisphenol AF and PPB, were added, and the mixture was processed for 10 min. The amounts of curing agents used in all formulations were 6 phr of calcium hydroxide, 3 phr of magnesium oxide, and 2 phr of bisphenol AF for all samples prepared. The filler content varied in proportions of 5, 10, 15, 20, and 25 phr. The nomenclature of the samples consisted of indicating the matrix (FKM), the type of filler used (GdO, GdB, or GdBT), and its corresponding proportion.

The conditions for the curing process were determined using a moving die rheometer, model ZL-3001, Zhongli Instrument Technology Co. Ltd. (Dongguan, China), at 175 °C, for 10 min. The curing of FKM-based composites was performed by using a laboratory hydraulic press with heated plates, model ZL-3022, Zhongli Instrument Technology Co. Ltd. (Dongguan, China). The selected conditions for crosslinking the FKM-based composites were 175 °C for 12 min. The composites underwent post-crosslinking processes in an oven, Memmert (Buchenbach, Germany), at 200 °C for 24 h to attain maximum physical properties.

### 2.4. Characterization of Powder Materials

The materials GdO, GdB, and GdBT were characterized using a model FT/IR-4X spectrometer, Jasco (Tokyo, Japan), for infrared analysis in attenuated total reflection mode (FTIR-ATR). Spectra were recorded in the range of 500 to 4000 cm^−1^. These materials were also characterized by Powder X-ray diffraction (PXRD) at room temperature on a Bruker D8 Advance diffractometer in Bragg-Bretano geometry, equipped with a CuKα radiation source in a range of 5° < 2θ < 80°. In addition, for particle size distribution, the powder materials were characterized by using a particle size analyzer 90Plus, Brookhaven (New York, NY, USA), using a solution of ethanol and the powders used as filler at 0.01 g/mL.

### 2.5. Characterization of Powder Fluoroelastomer Composites

The cure curves of the compounds were determined using a moving die rheometer (model ZL-3001, Zhongli Instrument Technology Co. Ltd., Dongguan, China) at 175 °C. The analysis was performed according to GB/T 16584 and ISO 6504 standards [21,22]. Approximately 4 g of material was weighed and placed in a rheometer chamber for 15 min at 175 °C; the oscillation arc was 1.0° and the cavity die frequency was 1.7 Hz. All specimens were measured in triplicate. The stress–strain test was determined using a Shimadzu EZ-X L 200 V instrument (Shimadzu, Kyoto, Japan) with a load cell of 500 N at 100 mm/min according to ASTM Standard (ASTM D412) [23].

The morphology of the failure points of FKM and FKM composite samples was analyzed using a GeminiSEM 360 Gemini 1 (Carl Zeiss, Oberkochen, Germany) field emission scanning electron microscope (FESEM).

Thermal properties were determined by thermogravimetric analysis (TGA) measurements using a Netzsch (Selb, Germany) model Iris TG 209 F1 thermogravimetric analyzer under a nitrogen atmosphere at a heating rate of 10 °C/min. The operating temperature range was set at 25 to 900 °C. Differential scanning calorimetry (DSC) of the different FKM composites was performed using a Netzsch DSC 214 (Selb, Germany) in the range between −100 °C and 25 °C with a heating rate of 10 °C/min.

Dynamic mechanical analysis (DMA) of FKM and FKM composites was performed using rectangle samples with a length of 40 mm, a width of 10 mm and a thickness of 2.5 mm. Amplitude sweep tests under shear deformations were performed at a constant frequency of 1 Hz (*ω* = 6.28 rad/s). The values of the shear strain were selected between 0.001 and 30%. The initial value for the extensional force was selected as small as 1 mN to ensure the measurement started well within the linear viscoelastic regime (LVR). The temperature was set at 20 °C. Temperature ramp tests under extensional deformation of 0.1% were performed at a constant frequency of 1 Hz (*ω* = 6.28 rad/s). The values of the ramp were selected between −50 °C and 40 °C.

The crosslink density was determined based on the theory of rubber elasticity. For this purpose, the rubber plateau value d E′ (Tg + 50 K) and the equations were used [24].

(1) $$\nu_{c}=\frac{E^{\prime }}{3RT}$$

(2) $$M_{c}=\frac{\rho }{\nu_{c}}$$

(3) $$E^{\prime }=\phi_{m}E_{C}$$

where $\nu_{c}$ is the crosslink density, $M_{c}$ is the average molecular weight between crosslinks, $E^{\prime }$ is the storage modulus of the matrix, ρ is the density of the matrix, $E_{C}$ is the storage modulus obtained from the rubbery plateau, $\phi_{m}$ is the volume fraction of the matrix in the composite. R and T are the gas constant (8.314 J mol^−1^ K^−1^) and the absolute temperature in K.

The surface free energy (SFE) of the samples was obtained by the following process. Firstly, the contact angles of each sample for two test liquids, deionized water and diiodomethane Reagent Plus (>99%, Sigma-Aldrich, St. Louis, MO，USA), were measured using an optic tensiometer (Theta Lite Attension Biolin Scientific, Gothenburg, Sweden) under atmospheric conditions at room temperature (20 ± 3 °C). The contact angle measurements for each liquid with 2 μL droplets were performedon samples prepared in sextuplicate under ambient conditions. The contact angles were obtained by analysis of pictures of the droplet taken during the 10 s after 5 s, after it had fallen on the sample surface. Thereafter, the SFEs for the samples were calculated based on the measured contact angles in accordance with Owens-Wendt theory, which can be expressed as follows:

(4) $$\sqrt{\gamma_{L1}^{d}\;\gamma_{S}^{d}}+\sqrt{\gamma_{L1}^{p}\;\gamma_{S}^{p}}=\frac{\gamma_{L1}^{total}\left(1+\cos\theta_{L1}\right)}{2}$$

(5) $$\sqrt{\gamma_{L2}^{d}\;\gamma_{S}^{d}}+\sqrt{\gamma_{L2}^{p}\;\gamma_{S}^{p}}=\frac{\gamma_{L2}^{total}\left(1+\cos\theta_{L2}\right)}{2}$$

(6) $$\gamma^{total}=\gamma^{p}+\gamma^{d}$$

where subscript indices L1 and L2 indicate the test liquids, water and diiodomethane, respectively; $\gamma^{total}$, $\gamma^{p}$, $\gamma^{d}$ are the area total SFE, polar (hydrogen) component of SFE, and dispersive component of SFE for the examined composites (or control samples), respectively; $\gamma_{L}^{total}$, $\gamma_{L}^{p}$ and $\gamma_{L}^{d}$ are the total SFE, polar component of SFE, and dispersive component of SFE of the test liquids (water and diiodomethane), respectively. The SFE of the test liquids used are the previously reported values by Pergal et al. [25]: water: $\gamma_{L1}^{total}=72.8\frac{mN}{m}(\mathrm{mJ}/m^{2})$, $\gamma_{L1}^{p}=51.0\frac{mN}{m}(\mathrm{mJ}/m^{2})$, $\gamma_{L1}^{d}=21.8\frac{mN}{m}(\mathrm{mJ}/m^{2})$; and diiodomethane $\gamma_{L2}^{total}=50.8\frac{mN}{m}(\mathrm{mJ}/m^{2})$, $\gamma_{L1}^{p}=0\;(\mathrm{mJ}/m^{2})$, $\gamma_{L1}^{d}=50.8\frac{mN}{m}(\mathrm{mJ}/m^{2})$; θ represents the measured contact angle for the test liquids. Contact angle measurements were performed at six random points on the FKM and FKM-based composite sample surfaces.

Solid-state UV spectra were recorded using Evolution 260 BIO (Thermo Scientific, Waltham, MA, USA) UV-Visible spectrophotometer coupled with an integration sphere. The bandgap (E_g_) values of the composites were determined from the Tauc plots obtained from the UV–vis diffuse reflectance spectra.

(7) $$\alpha h\nu =A{\left(h\nu -E_{g}\right)}^{n/2}$$

where h is Planck’s constant, *n* is the vibration frequency, a is the absorption coefficient, E_g_ is the band gap, and A is a proportionality constant. The value of the exponent n denotes the nature of the simple transition. For an indirect transition, n = 4. The α term in the Tauc equation was substituted with the Kubelka-Munk function, F(R_∞_).

## 3. Results and Discussion

**Figure 2:**
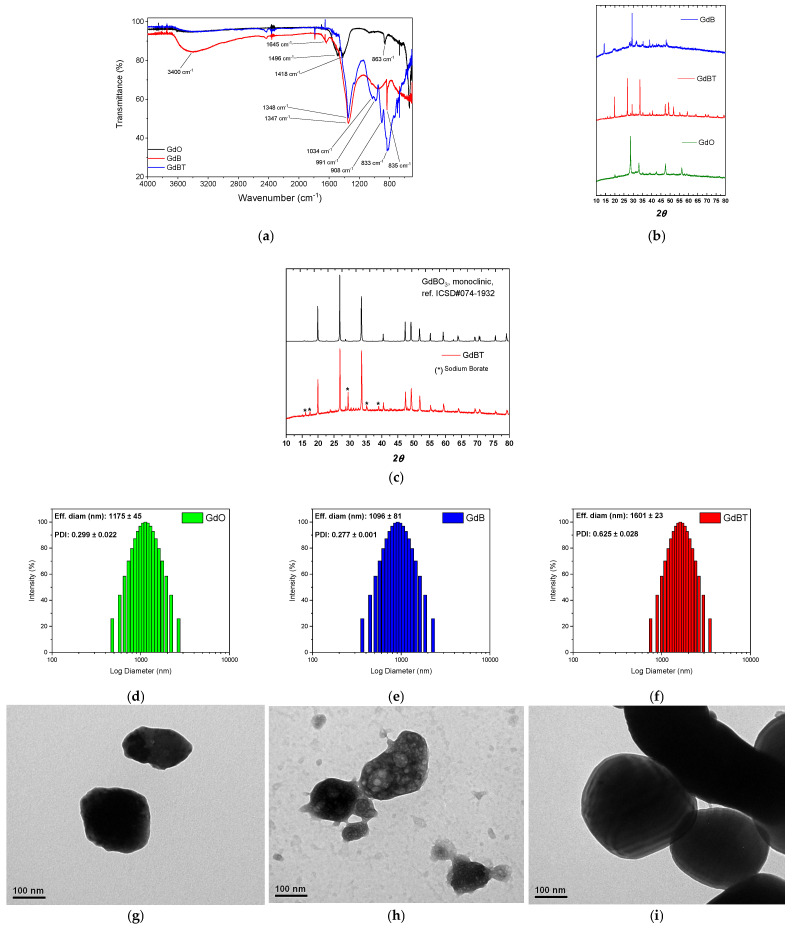
(**a**) FTIR spectra and (**b**) PXRD of gadolinium oxide, GdB and GdBT. (**c**) PXRDpatterns for GdBO_3_ monoclinic and comparative analysis for GdBT, where peaks of sodium borate are highlighted. Particle size distribution by dynamic light scattering (DLS of (**d**) GdO, (**e**) GdB and (**f**) GdBT. TEM images of (**g**) GdO, (**h**) GdB and (**i**) GdBT).

Figure 2a shows the FTIR spectra of the materials used as fillers in fluoroelastomer compounds. Significant evidence can be observed among the samples analyzed. For gadolinium(III) oxide, the peaks observed at 1496 cm^−1^ and 1418 cm^−1^ are characteristic of stretching vibrations of the carbonate ion (CO_3_^2−^). This indicates that the surface of the gadolinium oxide has adsorbed atmospheric CO_2_ or moisture, forming basic carbonates, a common phenomenon in rare earth oxides due to their hygroscopic nature [26]. Additional peaks at 1044 cm^−1^, 991 cm^−1^, and 908 cm^−1^ could be attributed to Gd-O bond vibrations in the presence of impurities or adsorbed hydroxyl groups, suggesting relative surface instability [27]. In contrast, the GdB spectrum shows a different pattern, with peaks at 1347 cm^−1^ and 1034 cm^−1^ corresponding to asymmetric and symmetric stretching vibrations of B-O bonds in BO_3_ groups, respectively, while the peaks at 883 cm^−1^ and 835 cm^−1^ are assigned to bending vibrations or deformation modes of the borate structure [28]. The absence of significant peaks above 1400 cm^−1^ in GdB indicates a lower presence of carbonates, suggesting that the preparation of GdB confers greater chemical stability against CO_2_ adsorption. In the case of GdBT, although no specific peaks are shown, it can be inferred that the heat treatment improved the crystallinity and purity of the material, possibly sharpening the existing bands and further reducing the adsorbed species, which would result in a more defined spectrum like that of GdBO_3_ but with better resolution of the characteristic borate peaks. Taken together, these results highlight that the synthesis of GdB and GdBT mitigates the sensitivity to surface contamination observed in GdO, and that additional heat treatment optimizes the structure of the material.

Moreover, the powder diffraction patterns of filler phases are shown in Figure 2b. The PXRD pattern of the GdBT phase shows narrow peaks, indicating that this sample has high crystallinity. In contrast, the PXRD pattern of the GdB phase displays broader peaks and a noisy baseline, suggesting that this sample has low crystallinity. The GdBT PXRD peaks are consistent with the GdBO_3_ crystal structure reported in ICSD#074-1932, which has a monoclinic crystal structure with C2/c space group (see Figure 2c). However, five peaks do not align with this monoclinic structure, suggesting that a secondary phase was formed during the thermal treatment. These secondary phase peaks can be attributed to sodium borate.

In order to identify the variation in the particle size distribution of GdO, GdB, and GdBT materials, a dynamic light scattering (DLS) analysis was performed, the results of which are shown in Figure 2d–f. All samples have a wide size distribution, falling within the microparticle range. The distribution of GdO is comparable in extent to that of GdB and GdBT. However, when comparing the latter two, it can be seen that GdBT has a larger average size than GdB. This difference is attributed to possible sintering processes that occurred during the heat treatment applied to GdBT.

On the other hand, Figure 2g–i also includes transmission electron microscopy (TEM) images of the three materials. These confirm that the particles have a low aspect ratio and high electron density, a consequence of the high atomic number of gadolinium (Gd). The GdB and GdBT particles maintain the low aspect ratio, but in the case of GdB, their electron density is irregular, suggesting the presence of light elements such as atoms from hydration water molecules. In the case of GdBT, a more homogeneous and higher electron density is observed, together with an evident agglomeration of particles, confirming that sintering has occurred because of heat treatment.

### 3.1. Curing, Mechanical and Dynamic Mechanical Properties of FKM-Based Composites

**Figure 3:**
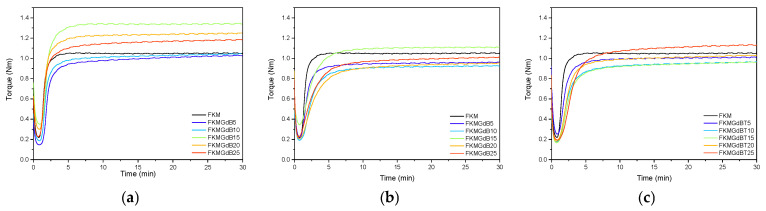
Curing curves of FKM nanocomposites containing GdO (**a**), GdB (**b**) and GdBT (**c**).

Table 1 shows the results of the rheological analysis performed on FKM formulations containing gadolinium(III) oxide, as well as gadolinium(III) borates (GdB and GdBT). Their corresponding curves are shown in Figure 3. The minimum modulus (M_L_), which is related to the viscosity of the compound and the polymer-filler interaction prior to vulcanization, exhibits varied behavior depending on the type of filler. Gadolinium oxide (GdO) significantly increases the minimum modulus, particularly at a 15 phr content. This indicates interaction between the polymer and the filler, which restricts the polymer’s flow. Conversely, GdB and GdBT modestly impact the minimum modules; their values tend to be similar to or lower than those of FKM, suggesting poor particle-polymer interaction. Regarding the maximum torque and the difference (M_H_–M_L_), which is proportional to the density of the crosslink network, a high value indicates a stiffer compound with a higher degree of crosslinking. GdO is observed to consistently increase the difference between M_H_ and M_L_, suggesting a higher crosslink density. This is especially noticeable in the FKM-GdO15 sample, which has the highest value: 0.973 Nm. This indicates that the filler actively participates in and promotes the efficiency of the vulcanization process. Additionally, GdO may act as a reinforcing filler. For GdB-based compounds, the M_H_–M_L_ difference decreases compared to FKM, indicating that GdB interferes with the crosslinking process [6,29]. GdBT shows intermediate M_H_–M_L_ difference values at low loads but similar values to those of GdB with 20 and 25 phr content.

Regarding the scorch time (t_s2_), GdO is observed to have a distinct effect compared to FKM. However, GdB and GdBT are found to have a significant effect, which could be associated with retarding vulcanization. Regarding the optimal vulcanization time (t_90_), which is related to the speed of forming the polymer cross-linked network, GdO slightly increases t_90_. For GdB- and GdBT-based composites, t_90_ values are longer, indicating a retarding effect on the curing process.

**Figure 4:**
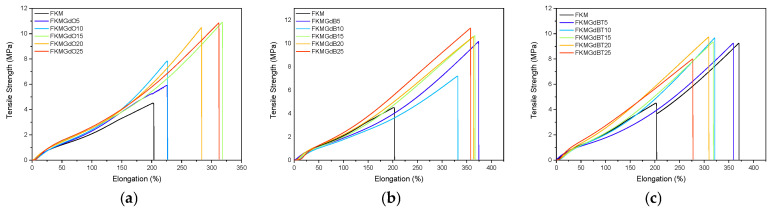
Stress–strain curves of neat FKM and FKM-based composites containing GdO (**a**), GdB (**b**) and GdBT (**c**) nanocomposites.

Table 2 shows the results of tensile tests on fluoroelastomer compounds containing different fillers. Representative tests for each composition are presented in Figure 4. There is a general trend whereby higher filler content increases the stiffness (elastic modulus) and tensile strength of the compounds, which indicates the effectiveness of the filler material as a reinforcing agent. It can be observed that some samples present optimum results; for instance, FKMGd15 has balanced properties, FKMGdB25 has the highest tensile strength, and FKMGdB5 has the highest extensibility. Moreover, significant differences can be seen between the various types of filler. For GdO, for example, both stiffness and tensile strength steadily increase with the amount of filler. This does not imply a decrease in elongation at break, which also tends to increase gradually. Even more remarkable behavior is observed in compounds with GdB: not only do they show a reinforcing effect, evidenced by the increase in modulus and strength, but they also demonstrate a substantial improvement in elongation at break—much higher than that observed in compounds with GdB and GdBT. Specifically, neat FKM has an elongation at break of 201%, whereas compounds with GdB can reach values of up to 370%. This unusual behavior, involving a simultaneous increase in tensile strength and elongation at break, suggests that GdB filler maximizes the mechanical performance of FKM compounds. The simultaneous improvement in tensile strength and elongation of FKM compounds with GdB is attributed to gadolinium’s multifunctional action. The availability of its f-orbitals enables the Gd^3+^ cation to form dynamic coordination bonds with the fluorine atoms in the polymer chains, creating a dual network in addition to the traditional covalent crosslinks [30,31,32].

Similarly, the presence of ${BO}_{3}^{3-}$ and hydration water molecules in the structure of GdB and GdBT gives these compounds a polar character that increases their affinity with the FKM fluoroelastomer. All of this could contribute to the formation of a reversible ionic network. This reversible ionic network dissipates energy under stress, thereby increasing toughness and elongation while also reinforcing the filler-polymer interface to improve stiffness and strength. Unlike the oxide in GdO, the borate anion in GdB appears to optimise this effect by promoting a more uniform and stable network. However, surface treatments in GdBT could inhibit these direct interactions, which could explain their lower effectiveness.

It is interesting to note that, in FKM-based compounds, the general tendency of fillers is to increase stiffness at the expense of a reduction in maximum elongation, i.e., a loss of elasticity. This behavior has been documented, for example, by Maldonado-Magnere et al. [6,33], who demonstrated that materials such as graphite, bismuth(III) oxide, and graphene nanoplatelets systematically lead to a decrease in elastomer ductility. However, the GdO, GdB, and GdBT systems studied here show a notable deviation from this trend. This change sheds light on the possibility of designing composites in which ionic interactions, favored by the polar nature of the FKM, play a fundamental role and contribute to better overall mechanical performance, simultaneously improving stiffness and toughness.

**Figure 5:**
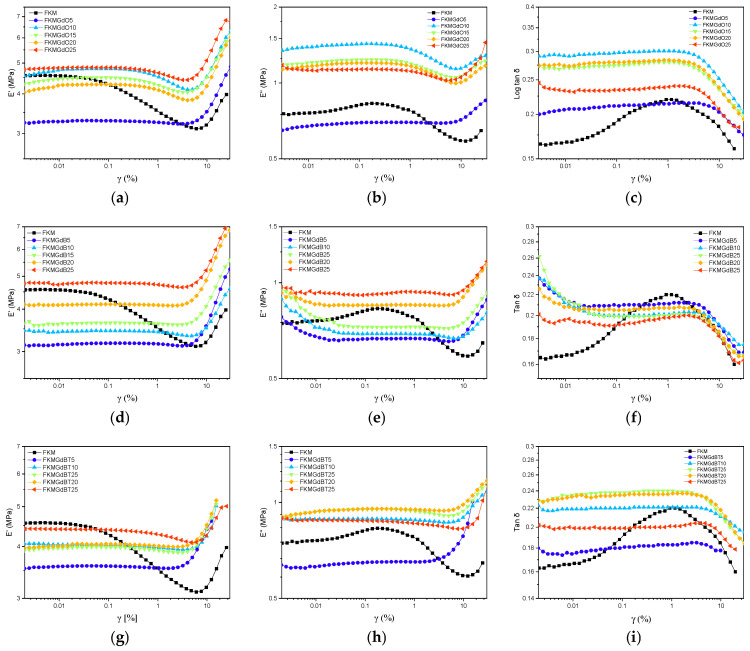
Dynamic mechanical parameters in shear strain mode of neat FKM and FKM composites consisting of storage modulus-loss modulus and loss factor of GdO based nanocomposites (**a**–**c**), GdB nanocomposites (**d**–**f**) and GdBT (**g**–**i**) nanocomposites.

Figure 5 shows the outcomes of the strain sweep test for FKM-based samples. It depicts how the storage modulus (E′), loss modulus (E″), and loss factor (tan δ) change with applied deformation. The dominant Payne effect is observed, which is characterized by a decrease in storage modulus (E′) as strain amplitude increases. This behavior is typical of elastomer compounds filled with fillers. In the low strain region, E′ remains high and constant, indicating that the network formed by the filler particles remains intact. As deformation increases, however, this network begins to break down, leading to a drastic decrease in E′. Regarding the loss modulus (E″), the unfilled FKM compound exhibits a broad, low-intensity peak in the nonlinear region, which is related to the internal friction of the polymer network. When filler is incorporated (from 5 to 25 phr), the height and shape of the E″ curve change noticeably, and a much more pronounced peak appears. This suggests that the filler introduces a new energy dissipation mechanism associated with its network breakdown, which is linked to greater material hysteresis [34,35]. Regarding the loss factor (tan δ), a maximum peak is also observed in the nonlinear deformation region. Lower values of the base FKM are associated with more elastic and less dissipative behavior. Adding filler causes a drastic increase in the magnitude of this peak, implying that the filler introduces additional energy dissipation mechanisms.

The behavior of compounds with GdB filler is completely opposite. At low deformations, the storage modulus (E′) is similar to or lower than that of FKM without filler, indicating that this filler does not reinforce the polymer matrix. Considering the lower ΔM (MH-ML) values obtained from the curing curves, active interference with the crosslinking process can be inferred, resulting in lower crosslink density. The minimal decrease in E′ with increasing deformation suggests a weak or nonexistent Payne effect, confirming the absence of a percolated filler network. Regarding the loss modulus (E″), the absence of a defined peak corroborates the lack of a significant filler structure capable of breaking and releasing energy in a dissipative manner under deformation. This implies that energy dissipation is solely dominated by the weakened polymer network [36,37]. Regarding tan δ, a flat, linear trend is observed in the compounds, suggesting linear viscoelastic behavior associated with very low hysteresis.

For FKM samples with treated filler content (GdBT), intermediate behavior is observed. The storage modulus (E′) at low deformation is higher than that of the compound with untreated GdB and the base FKM but lower than that of the compounds with GdB. This indicates that the treatment gave the filler a moderate reinforcing capacity. Although less pronounced than in the GdO series, a decrease in E′ with deformation (Payne effect) is observed, indicating the formation of a filler network that is less rigid or extensive [36,38,39]. Conversely, the appearance of a discernible peak in the loss modulus curve (E″) suggests the presence of energy dissipation processes associated with the rupture of the reformed filler network. The delta tangent (tan δ) values show a moderate increase compared to the base FKM, indicating an improved balance between elastic and dissipative properties compared to untreated GdB. This improvement is thanks to the treatment applied to the filler.

**Figure 6:**
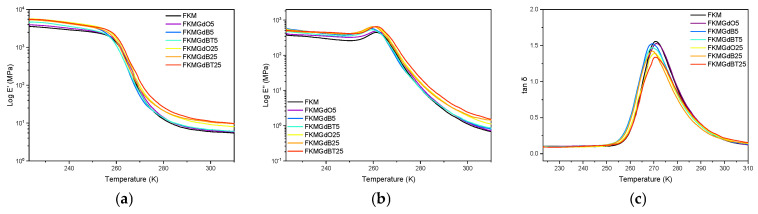
Dynamic mechanical parameters under a temperature ramp of neat FKM and FKM composites, consisting of (**a**) storage modulus; (**b**) loss modulus; and (**c**) loss factor.

Analyzing viscoelastic parameters such as the storage modulus (E′), the loss modulus (E″), and the delta tangent (tan δ) as a function of temperature at a constant frequency allows one to characterize the transition of a material from a rigid glassy state to an elastic rubbery state. Figure 6 shows the behavior of the storage modulus (E′). All materials exhibit the abrupt drop characteristic of this transition. However, composites with a higher filler content (25 phr) exhibit a less pronounced decrease in E′ and higher absolute values in the rubbery plateau compared to the unfilled elastomer. This indicates a reinforcing effect: the filler particles restrict the movement of polymer chains, increasing the stiffness of the composite material. The loss modulus (E″) increases slightly in compounds with higher filler content, suggesting that the filler particles affect dissipative processes across the analyzed temperature range [40].

The peak of the delta tangent (tan δ) was analyzed using a nonlinear curve fit for an asymmetric peak to determine the transition temperature (T_g_), full width at half maximum (FWHM), and maximum height [41]. The T_g_ values corresponding to these peaks’ maximums show that adding GdB fillers (at both concentrations) and GdBT at 5 phr significantly decreases T_g_, from −2.14 °C (FKM) to −3.62 °C. This decrease suggests a possible plasticizing effect in which the particles interfere with the polymer’s intermolecular interactions, facilitating the movement of chain segments at lower temperatures [42,43]. Regarding network homogeneity, the FWHM value of FKM0 (17.2 °C) increases in all compounds, reaching values up to 18.5 °C. This broadening indicates that the incorporation of fillers introduces heterogeneity to the system because the fillers create interfaces with the polymer matrix that have different molecular mobility and activation energy than the polymer in the solid state [44,45,46]. Finally, the maximum height of the tan δ peak, which is related to the material’s ability to dissipate energy, decreases significantly in formulations with a high filler content (25 phr). This confirms that a greater amount of filler particles more effectively restricts chain movement, reducing the magnitude of relaxation and consequently the damping capacity of the material in the glass transition region.

Based on data from the storage module (E′) in the gummy-state plateau, the effective crosslink density of the polymer network was determined, and it was observed that the incorporation of gadolinium-based fillers promotes a significant increase in this density, which reaches its maximum value for the FKMGdBT25 compound. This increase suggests that these fillers actively promote network formation through a dual mechanism: on the one hand, their chemical nature could catalyze the covalent crosslinking reaction promoted by bisphenol AF and, on the other hand, they establish ionic interactions with the polar FKM, forming a dynamic network of supplementary physical bonds. This reinforcement mechanism through specific interactions between the filler surface and the elastomer, widely documented in the specialized literature (Table 3) [24], manifests itself in an increase in the calculated effective crosslink density and explains the exceptional balance of mechanical properties observed in these compounds.

### 3.2. Morphology of Fluoroelastomers Based Composites

**Figure 7:**
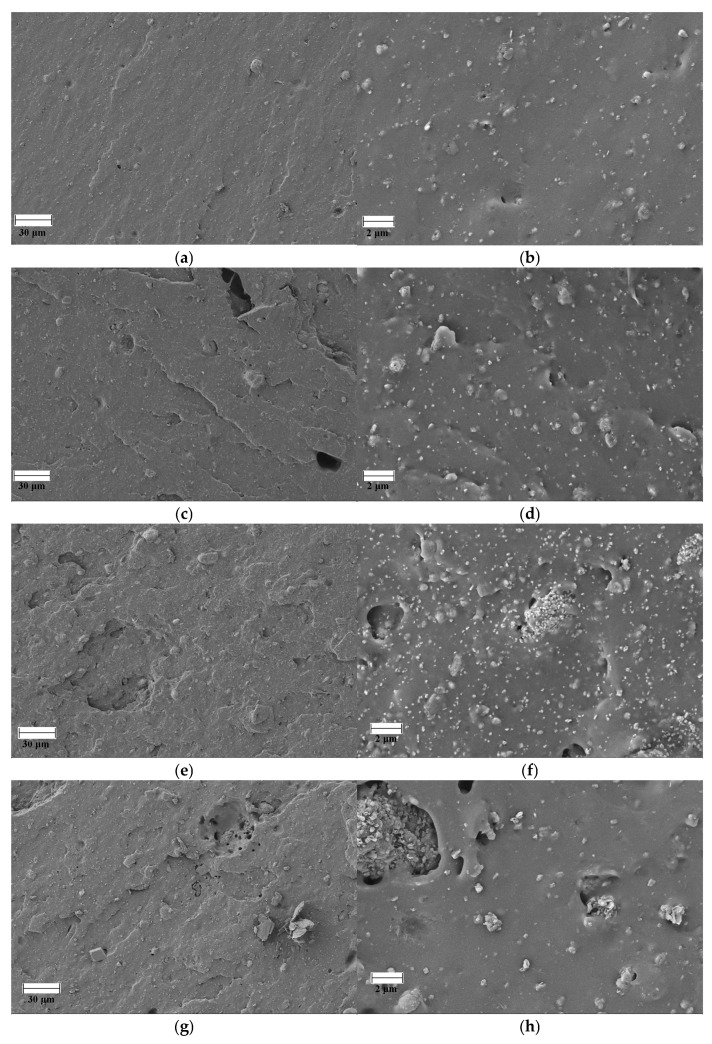

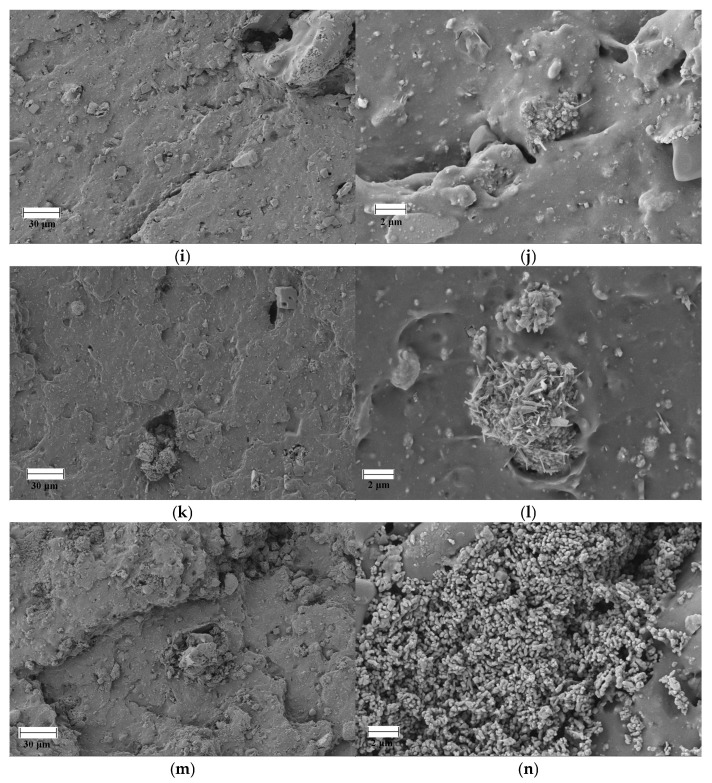
SEM images of FKM (**a**,**b**), FKMGdO5 (**c**,**d**), FKMGdO25 (**e**,**f**), FKMGdB5 (**g**,**h**), FKMGdB25 (**i**,**j**), FKMGdBT5 (**k**,**l**) and FKMGdBT25 (**m**,**n**).

Figure 7 shows SEM images of the failure points of the specimens tested by tensile testing of FKM and FKM compounds loaded with GdO, GdB, and GdBT. Neat FKM has a morphology typical of crosslinked rubber, where the continuous elastomer phase contains particles of crosslinking agents based on the bisphenol AF system, such as magnesium oxide and calcium hydroxide. The presence of some bubbles that can be observed is mainly due to the release of volatile compounds during the cross-linking reaction. These compounds, which may be by-products of the decomposition of curing agents or residual moisture, become trapped in the polymer matrix when the curing kinetics and the increase in viscosity exceed their ability to diffuse to the surface. The incorporation of gadolinium(III) oxide as a filler introduces significant morphological changes. At low concentrations (5 phr), the filler affects the continuity of the phase, evidenced by a greater number of fracture surfaces, suggesting the formation of failure points initiated by the particles. However, the dispersion of the filler in this case appears to be homogeneous. In contrast, the FKMGdO25 compound not only exhibits these fracture zones, but also greater agglomeration of the filler material. In the FKMGdB and FKMGdBT compounds, agglomeration of the filler is observed in all concentrations analyzed. Similarly, it is possible to distinguish heterogeneity associated with the type of filler, where a morphological contribution corresponding to gadolinium(III) oxide and another to sodium borate is identified, both materials being distributed randomly within the elastomer matrix. The results obtained indicate that, despite the high agglomeration of the filler particles, a significant improvement in mechanical properties is achieved. This suggests that their contribution is not limited solely to the stiffness effect imparted by a rigid load, as might be expected. As discussed, the superior performance of these materials is mainly due to the formation of a dynamic network mediated by ionic interactions between the particles, which acts as an additional and decisive reinforcement mechanism.

### 3.3. Thermogravimetric Analysis of Fillers and FKM Gadolinium Composites

**Figure 8:**
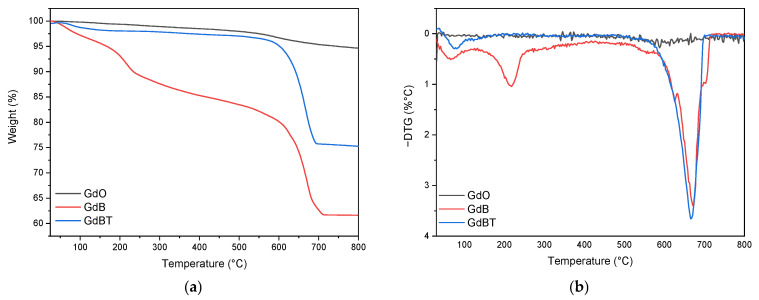
Thermogravimetric analysis (**a**) and derivative curve of thermogravimetric analysis (**b**) of GdO, GdB and GdBT.

Figure 8 shows the thermogravimetric analysis (TGA) of the materials used as fillers: GdO, GdB, and GdBT. The same figure includes the derived thermogravimetric curve (DTG), which identifies the different decomposition peaks for each sample.

The GdO sample shows a slight peak around 600 °C, suggesting the decomposition of residual impurities, such as gadolinium(III) hydroxide or carbonate. The GdB and GdBT samples, on the other hand, show several degradation processes. In the case of GdB, three events are observed: the first around 100 °C, associated with adsorbed moisture; the second near 218 °C, attributed to the dehydration of the material; and the third, a pronounced peak at 673 °C. The latter is related to a significant loss of mass due to the decomposition of sodium borate, which involves a complex process of dehydration, amorphization, and crystallization, with the elimination of hydroxyl groups in the form of water [47]. As for GdBT, signals corresponding to moisture loss and the process associated with sodium borate are detected. The absence of the dehydration peak around 218 °C suggests a higher degree of crystalline ordering in this material, which coincides with what was observed by X-ray diffraction.

**Figure 9:**
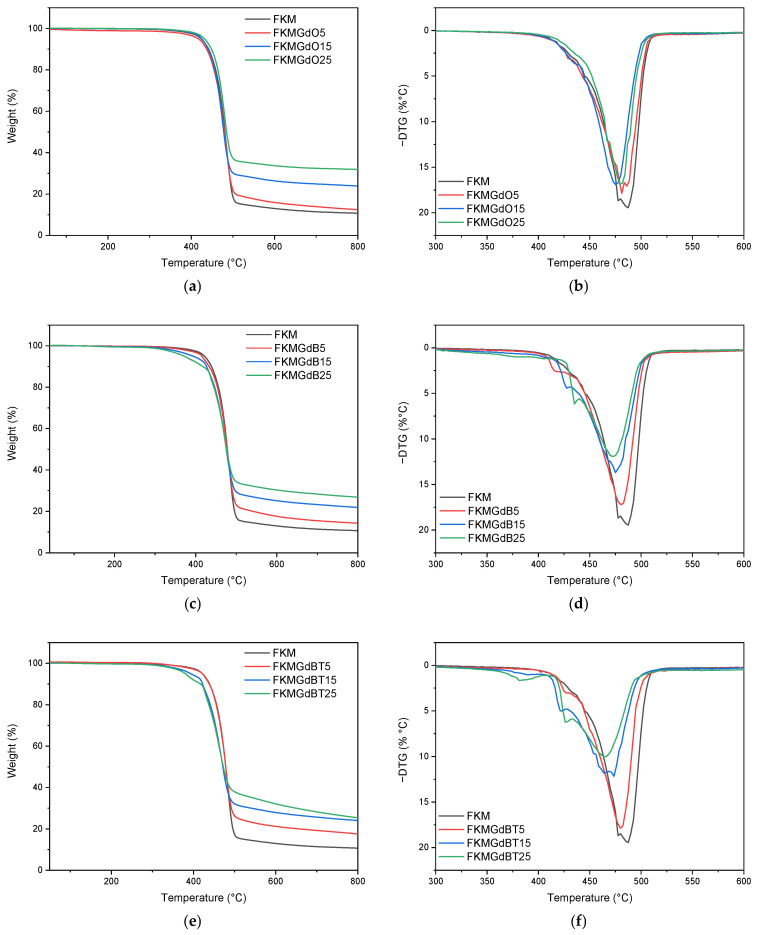
Thermogravimetric analysis and derivative curve of thermogravimetric analysis of FKMGdO (**a**,**b**), FKMGdB (**c**,**d**) and FKMGdBT (**e**,**f**) composites.

Regarding the thermogravimetric analysis of FKM compounds, Figure 9 shows the curves for compounds with different GdO, GdB, and GdBT contents. In FKM compounds with GdO, a single degradation process attributable to the decomposition of the polymer matrix is observed. Analysis of the derivative curve (DTG) reveals that the maximum degradation ratio temperature of pure FKM is 487 °C. This value shifts to lower temperatures, 481 °C, 475 °C, and 479 °C for the FKMGdO5, FKMGdO15, and FKMGdO25 samples, respectively, suggesting a decrease in thermal stability. This phenomenon could be due to a catalytic effect of the gadolinium oxide (GdO) surface, which reduces the activation energy required for polymer degradation. The initial and progressive decrease in the DTG peak for the 5 and 15 phr contents, followed by a slight increase at 25 phr, indicates that at low concentrations, optimal dispersion is achieved, maximizing the catalytic effect. Conversely, at higher contents (25 phr), particle agglomeration reduces the surface effectiveness of GdO.

For their part, FKM compounds with GdB also exhibit a single main decomposition process. However, the DTG curve shows an early onset of degradation, visible as a shoulder at a lower temperature adjacent to the main peak. The size of this shoulder is proportional to the GdB content, suggesting that it is associated with early decomposition of the filler material itself. In addition, a decrease in the temperature of the main DTG peak is observed at 480 °C, 475 °C, and 473 °C for the FKMGdB5, FKMGdB15, and FKMGdB25 samples, respectively, confirming that the presence of gadolinium promotes earlier degradation of the polymer chains. Finally, the FKMGdBT5, FKMGdBT15, and FKMGdBT25 compounds show a similar trend to that of the compounds with GdB, with DTG peaks recorded at 480 °C, 474 °C, and 466 °C, respectively, which shows a similar influence of gadolinium on the thermal degradation of the elastomer.

**Figure 10:**
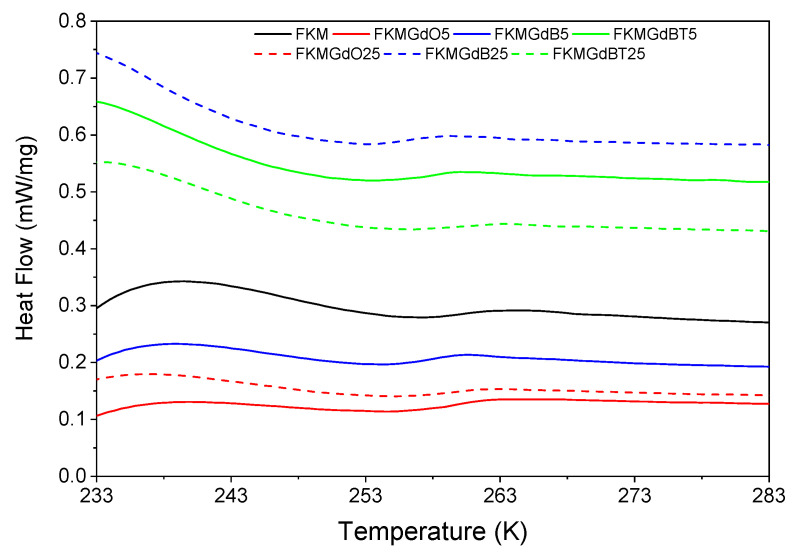
Differential calorimetry analysis of FKM-based composites.

In the differential scanning calorimetry (DSC) analysis shown in Figure 10, unfilled FKM has a glass transition temperature (T_g_) of −13.7 °C. However, compounds incorporating GdO, GdB, and GdBT fillers exhibit lower T_g_ values. Specifically, for samples with 25 phr of filler, FKMGdO25, FKMGdB25, and FKMGdBT25 record temperatures of −14.9 °C, −17.0 °C, and −13.5 °C, respectively (Table 4). This general decrease suggests that the filler particles introduce free volume into the polymer matrix, which facilitates the segmental movements of the FKM chains and, consequently, reduces the temperature at which the material transitions from the glassy state to the rubbery state. This effect can be attributed to a polymer-filler interaction that does not significantly restrict the mobility of the chains and may even act similarly to a plasticizing effect.

### 3.4. Surface Energy and Band Gap of Nanocomposites

Table 5 shows the total surface energy and its components, dispersive and polar, determined using the Owens, Wendt, Rabel, and Kaelble (OWRK) method. Unfilled FKM has low total surface energy (25.9 mJ/m^2^), dominated by the dispersive component (24.1 mJ/m^2^) and with a minimal polar component (1.8 mJ/m^2^), which is characteristic of fluorinated elastomers and confirms their marked hydrophobic nature. With the addition of 5 phr of fillers (GdO, GdB, and GdBT), the total surface energy increases. This increase is directly attributed to a significant increase in the polar component in the compounds, indicating a more hydrophilic surface with greater potential for adhesive interactions. The significant increase in the polar component of surface energy observed in compounds containing 5 phr of filler indicates effective particle dispersion at low concentrations. When gadolinium oxide or borate particles are homogeneously distributed and do not agglomerate in the FKM, a large proportion of their specific surface area—intrinsically polar due to their ionic nature (Gd^3+^, O^2−^, BO_3_^3−^)—is exposed, creating an extensive and active interface with the polymer. This well-distributed interface introduces numerous new ‘polar sites’ on the surface of the compound, resulting in an increase in measured polar energy (γ_sp_). In contrast, at 25 phr, the tendency to agglomerate drastically reduces this effective interface; the polar surfaces of the particles are ‘hidden’ within the agglomerates and mainly contact each other. Consequently, the surface chemistry is again dominated by the non-polar regions of the FKM, resulting in a decrease in the polar component. Therefore, dispersion controls access to the polar groups of the filler, which has a profound effect on the interfacial and adhesion properties of the material.

**Figure 11:**
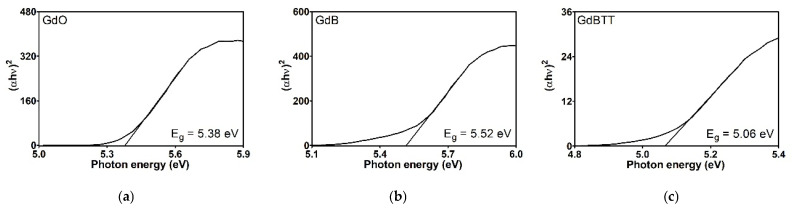
Tauc graphics of fillers: gadolinium oxide (**a**), gadolinium GdB mixture (**b**) and gadolinium GdBT mixture (**c**).

**Figure 12:**
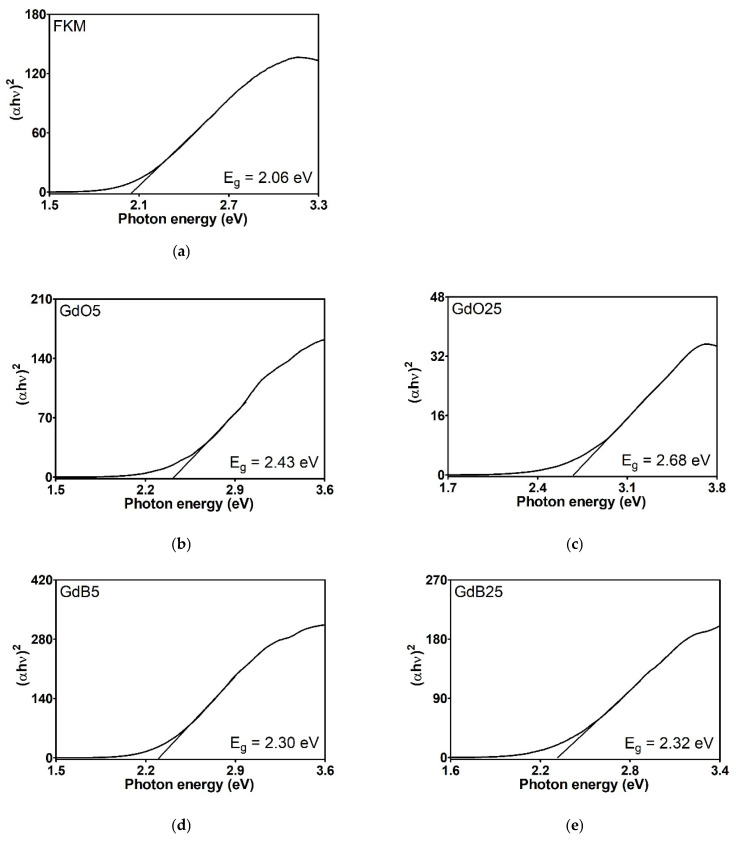

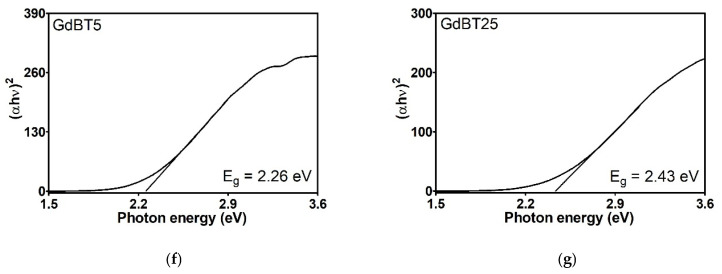
Tauc graphics of neat FKM (**a**) and FKM nanocomposites based on GdO (**b**,**c**), GdB (**d**,**e**) and GdBT (**f**,**g**).

The energy gap (E_g_), as determined by the Kubelka-Munk transformation, provides essential information regarding the electronic properties of materials, particularly the energy necessary to transition electrons from the valence band to the conduction band. The Eg values obtained for gadolinium-based fillers (Figure 11) are rather comparable. Nevertheless, FKM gadolinium-filled compounds (Figure 12) vary significantly, reflecting the impact of these additives on the material’s electronic structure. Neat FKM has a band gap of 2.06 electron volts (eV), placing it within the characteristic range of organic semiconductors. This value indicates moderate electrical conductivity, consistent with the nature of fluorinated elastomers. However, when gadolinium fillers (GdO, GdB, and GdBT) are added, a systematic increase in E_g_ is observed in all compounds. This widening of the band gap suggests that the materials become more insulating; that is, more energy is required to excite the electrons, thereby reducing the inherent electrical conductivity of FKM. Among the compounds with 5 phr of filler, GdO produces the largest increase (E_g_ = 2.43 eV), followed by GdB (2.30 eV) and GdT (2.26 eV). This indicates that GdO is the most effective at modifying the electronic structure of FKM at low concentrations, possibly due to stronger interactions at the polymer-filler interface that restrict the mobility of charge carriers. In compounds with 25 phr, the band gap continues to increase with GdO (2.68 eV) and GdBT (2.43 eV), while the increase is minimal with GdB (2.32 eV). This trend suggests that GdO and GdBT maintain their effectiveness in widening the gap at higher concentrations, probably due to homogeneous dispersion that favors the formation of interfaces altering the energy bands. However, GdB shows a saturation effect that could be related to agglomeration at high concentrations, limiting its interaction with the polymer matrix.

The general increase in E_g_ with the addition of fillers can be attributed to several factors. First, gadolinium oxides are materials with wide band gaps (e.g., pure GdO has an E_g_ of approximately 5 eV), so incorporating them introduces electronic states that widen the compound’s effective gap. Second, particle dispersion in the polymer matrix can affect chain organization, favoring a more amorphous structure that increases E_g_. Finally, interfaces between FKM and fillers can create energy barriers that hinder electronic transitions.

In conclusion, gadolinium fillers, particularly GdO, favorably modify the electronic properties of FKM, causing it to behave more like an insulator. This is advantageous for applications requiring high electrical resistance, such as coatings or seals in electrified environments. The dependence of E_g_ on filler type and concentration suggests the potential to tune the material’s electronic properties for specific applications.

## 4. Conclusions

This study demonstrated that it is possible to use materials such as gadolinium oxide and gadolinium borates in an FKM-type fluoroelastomer without impairing its curing properties. In addition, the remarkable mechanical properties observed—with increases of up to 162% in tensile strength and an almost twofold increase in elongation at break—represent an exceptional balance of properties that would allow these materials to be used in special applications. The mechanical-dynamic properties indicate that the nature of these fillers is not reinforcing, as the Payne effect is not observed, especially in compounds containing gadolinium borates.

Likewise, it was found that the incorporation of gadolinium-based materials influences the photophysical properties of the elastomer, showing an increase in the band gap that could be associated with a reduction in the inherent electrical conductivity of the material.

Taken together, these results represent a significant advance in the development of advanced materials that could find applications in radiation shielding, given the critical importance of boron and gadolinium for this function. These aspects are currently under investigation and will be reported later.

## Figures and Tables

**Table 1:** Curing parameters of FKM nanocomposites.

Composite	t_s2_ (min:sec)	t_90_ (min:sec)	M_L_ (Nm)	M_H_ (Nm)	M_H_–M_L_ (Nm)
FKM0	1:18 ± 0:02	3:13 ± 0:59	0.1825 ± 0.052	0.988 ± 0.095	0.806
FKMGdO5	1:42 ± 0:01	4:38 ± 0:29	0.149 ± 0.004	1.022 ± 0.013	0.873
FKMGdO10	1:32 ± 0:00	4:05 ± 0:07	0.182 ± 0.007	1.041 ± 0.008	0.859
FKMGdO15	1:38 ± 0:02	3:45 ± 0:14	0.334 ± 0.022	1.307 ± 0.057	0.973
FKMGdO20	1:22 ± 0:02	3:53 ± 0:09	0.250 ± 0.071	1.181 ± 0.099	0.931
FKMGdO25	1:20 ± 0:01	4:26 ± 0:04	0.225 ± 0.004	1.151 ± 0.051	0.926
FKMGdB5	1:30 ± 0:01	3:49 ± 0:05	0.199 ± 0.021	0.951 ± 0.019	0.752
FKMGdB10	1:51 ± 0:01	5:34 ± 0:07	0.187 ± 0.040	0.919 ± 0.013	0.732
FKMGdB15	1:43 ± 0:02	6:16 ± 0:44	0.199 ± 0.010	0.943 ± 0.052	0.744
FKMGdB20	2:00 ± 0:07	8:17 ± 1:02	0.205 ± 0.006	0.931 ± 0.025	0.726
FKMGdB25	1:40 ± 0:02	6:31 ± 0:21	0.227 ± 0.003	0.994 ± 0.018	0.767
FKMGdBT5	1:36 ± 0:07	5:02 ± 0:56	0.213 ± 0.045	0.971 ± 0.053	0.758
FKMGdBT10	1:42 ± 0:01	5:31 ± 0:33	0.175 ± 0.003	0.940 ± 0.039	0.765
FKMGdBT15	1:55 ± 0:03	6:50 ± 0:44	0.175 ± 0.011	0.957 ± 0.010	0.782
FKMGdBT20	1:44 ± 0:00	5:30 ± 0:17	0.179 ± 0.004	1.029 ± 0.004	0.850
FKMGdBT25	2:00 ± 0:12	7:05 ± 0:35	0.203 ± 0.012	1.151 ± 0.023	0.948

**Table 2:** Mechanical properties determined by tensile testing of FKM nanocomposites.

Composites	E50 (MPa)	E100 (MPa)	E200 (MPa)	E300 (MPa)	Tensile Strength (MPa)	Elongation at Break (%)
FKM0	1.139 ± 0.015	1.948 ± 0.032	4.245 ± 0.043	-	4.325 ± 0.081	201 ± 5
FKMGdO5	1.142 ± 0.083	2.091 ± 0.119	5.008 ± 0.074	-	5.897 ± 0.458	221 ± 6
FKMGdO10	1.238 ± 0.008	2.271 ± 0.005	5.974 ± 0.128	-	7.483 ± 0.123	234 ± 11
FKMGdO15	1.397 ± 0.057	2.384 ± 0.054	5.112 ± 0.093	9.496 ± 0.444	10.325 ± 0.533	312 ± 9
FKMGdO20	1.456 ± 0.022	2.542 ± 0.021	5.640 ± 0.051	-	10.049 ± 0.422	288 ± 3
FKMGdO25	1.470 ± 0.016	2.631 ± 0.017	5.939 ± 0.154	-	10.943 ± 0.750	292 ± 11
FKMGdB5	0.973 ± 0.013	1.683 ± 0.022	3.588 ± 0.086	6.760 ± 0.373	10.015 ± 0.181	386 ± 4
FKMGdB10	1.017 ± 0.010	1.762 ± 0.024	3.637 ± 0.051	6.421 ± 0.118	7.594 ± 0.124	321 ± 13
FKMGdB15	1.126 ± 0.008	1.971 ± 0.023	4.412 ± 0.171	7.888 ± 0.299	10.750 ± 0.417	381 ± 16
FKMGdB20	1.193 ± 0.039	2.103 ± 0.054	4.701 ± 0.114	8.143 ± 0.161	10.667 ± 0.324	372 ± 9
FKMGdB25	1.339 ± 0.038	2.396 ± 0.059	5.402 ± 0.252	9.023 ± 0.400	11.313 ± 0.244	370 ± 15
FKMGdBT5	1.013 ± 0.012	1.702 ± 0.011	3.689 ± 0.115	6.795 ± 0.338	9.433 ± 0.364	371 ± 2
FKMGdBT10	1.134 ± 0.025	1.970 ± 0.059	4.580 ± 0.129	8.284 ± 0.249	9.580 ± 0.130	326 ± 10
FKMGdBT15	1.246 ± 0.035	2.245 ± 0.089	5.139 ± 0.073	8.713 ± 0.202	9.229 ± 0.179	316 ± 8
FKMGdBT20	1.389 ± 0.036	2.577 ± 0.037	5.673 ± 0.108	9.295 ± 0.360	9.638 ± 0.445	310 ± 5
FKMGdBT25	1.481 ± 0.062	2.675 ± 0.100	5.621 ± 0.111	-	8.122 ± 0.271	285 ± 8

**Table 3:** Parameters such as glass transition temperature, FWHM and maximum height determined by analyzing the tan d peak of FKM-based composites. Crosslink density and average molecular weight between crosslinks.

Sample	T_g_-DMA (°C)	FWHM (°C)	Maximum Height	$\upsilon_{c}$ (mol/m^3^)	$M_{c}$ (g/mol)
FKM0	−2.14	17.2	1.41	699	2615
FKMGdO5	−2.14	17.2	1.42	725	2560
FKMGdB5	−3.62	18.1	1.42	766	2444
FKMGdBT5	−3.62	17.9	1.36	750	2448
FKMGdO25	−2.63	18.0	1.26	954	2217
FKMGdB25	−3.62	17.4	1.29	1042	1854
FKMGdBT25	−2.63	18.5	1.21	1080	1858

**Table 4:** Glass transition temperature of FKM-based composites determined by DSC.

Sample	FKM	FKMGdO5	FKMGdB5	FKMGdBT5	FKMGdO25	FKMGdB25	FKMGdBT25
T_g_	−13.7 ± 0.9	−14.6 ± 0.8	−15.5 ± 0.9	−16.0 ± 0.8	−14.9 ± 0.1	−17.0 ± 0.3	−13.5 ± 0.3

**Table 5:** Total surface $\gamma_{T}$, dispersive $\gamma_{D}$, and polar $\gamma_{D}$ energies of FKM-based composites.

Sample	$\gamma_{D}$ (mJ/m^2^)	$\gamma_{P}$ (mJ/m^2^)	$\gamma_{T}$ (mJ/m^2^)	E_g_ (eV)
FKM	24.1 ± 1.8	1.8 ± 0.5	25.9 ± 1.5	2.06
FKMGdO5	22.2 ± 1.1	4.7 ± 2.9	26.9 ± 2.1	2.43
FKMGdB5	24.3 ± 2.8	5.1 ± 1.1	30.3 ± 1.4	2.30
FKMGdBT5	23.2 ± 1.6	6.7 ± 1.6	29.9 ± 2.1	2.26
FKMGdO25	20.0 ± 3.1	2.1 ± 1.7	22.1 ± 1.7	2.68
FKMGdB25	19.3 ± 0.7	1.5 ± 0.9	20.8 ± 0.2	2.32
FKMGdBT25	22.6 ± 1.0	2.0 ± 0.4	24.6 ± 1.1	2.43

## Data Availability

The original contributions presented in the study are included in the article, further inquiries can be directed to the corresponding authors.
